# Surveillance of non-communicable diseases: What matters to users? A qualitative interview study

**DOI:** 10.25646/12919

**Published:** 2024-12-18

**Authors:** Robyn Kettlitz, Maike Buchmann, Oktay Tuncer, Laura Krause, Thomas Ziese, Lukas Reitzle

**Affiliations:** 1 Helmholtz Centre for Infection Research, Department of Epidemiology, Braunschweig, Germany; 2 PhD Programme ‘Epidemiology’, Braunschweig-Hannover, Germany; 3 Formerly Robert Koch Institute, Department of Epidemiology and Health Monitoring, Berlin, Germany; 4 Robert Koch Institute, Department of Epidemiology and Health Monitoring, Berlin, Germany

**Keywords:** NCD surveillance, Diabetes surveillance, Qualitative interviews, Information transfer, User evaluation

## Abstract

**Background:**

Surveillance systems for diseases serve as an early warning system and orientation for decision-makers. As part of the National Diabetes Surveillance at the Robert Koch Institute (RKI), existing formats of information transfer were evaluated and an analysis of users’ requirements regarding the dissemination of results of surveillance for non-communicable diseases (NCD) was carried out.

**Methods:**

13 semi-structured guided interviews were conducted with persons from health politics, healthcare, media and science and analysed in a qualitative content analysis (interview survey period: 10/2022 – 01/2023).

**Results:**

For all respondents, the frequency of diseases and their determinants, care and consequences were the focus of NCD surveillance. Wider determinants of health and illness situation were also considered relevant. Requirements regarding the presentation of these contents differed between the user groups. Factors that facilitate and inhibit the use of NCD surveillance information were consistent across the user groups.

**Conclusions:**

There is a need for the presentation of options for action, especially for users involved in health politics and healthcare. Diabetes surveillance showed that many requirements are already met by the existing formats. Many of the users also wanted the content to be expanded to include other NCD.

## 1. Introduction

Non-communicable diseases (NCD), such as diabetes mellitus, cardiovascular diseases, cancer and chronic respiratory diseases, lead to chronic health restrictions and are among the main causes of death worldwide [1, 2]. Many risk factors for NCD are preventable and according to the World Health Organisation’s (WHO) action plan for the prevention of NCD, surveillance is an important tool [3]. Surveillance is the continuous, systematic collection, analysis and interpretation of health-related data [4, 5]. It serves as an early warning system and orientation for decision-makers in the healthcare system and can support the evaluation of public health measures [4]. Ways of distributing results (hereafter dissemination) include websites, scientific publications, reports, presentations at specialist conferences, brochures and social media. The aim of dissemination is to make scientific findings accessible for decision-making processes and to align the presentation with the needs of the users [6, 7]. A lack of consideration for the requirements of users and limited financial resources and personnel, among other factors, can lead to the knowledge transfer being inefficient [8]. The relationship between politics and science can be strengthened by ensuring that data is available and results are up-to-date, accessible and reliable [9].

In 2015, the Robert Koch Institute (RKI) was commissioned by the Federal Ministry of Health (BMG) to develop the National Diabetes Surveillance. In the current fourth project phase, the expansion to NCD surveillance is taking place [6, 10–12]. The aim is to bundle existing surveillance activities for NCD in a centralised system. The project team, which comprises scientific staff from the units of *physical health* and *health monitoring*, developed a dissemination strategy together with the scientific advisory board of the diabetes surveillance, which entails four formats [6]: The *report* ‘*Diabetes in Germany*’ was designed for actors in health politics and healthcare [13]. For the entire professional community, a *website* (www.diabsurv.rki.de) was developed that interactively visualizes all indicators. The website also provides links to current information about the project, as well as *scientific publications* produced as part of the project, in which methods, data sources and results of the surveillance are presented, and further literature. In addition, *press releases*, and *social media* are used to draw the attention of the media to the results of diabetes surveillance [6].

The aim of the study described below was to identify the requirements of NCD surveillance users for the further development of formats and the dissemination strategy [6]. The following questions were to be answered:

1. What information do users need?
2. What are the requirements for content and formats?
3. Which criteria inhibit or facilitate use?
4. What experiences have the respondents already had with the diabetes surveillance?

## 2. Methods

### 2.1 Data collection

Semi-structured interviews were conducted with experts from the field of health to determine the needs of NCD surveillance users. Questions on everyday professional life and search behaviour, as well as requirements for NCD topics, formats, information quality and data preparation were part of the interview. Participants who had already used diabetes surveillance formats were asked about their experiences. Finally, the profession and years of experience in the current field of work were asked. The questionnaire was checked for comprehensibility and accuracy by the study team prior to the interviews.

The selection and invitation was conducted according to the ‘purposive sampling’ [14–16] i.e. people who use data or information on the health situation of the population in Germany for professional reasons were purposively recruited in a targeted manner. People who worked in the fields of health politics, healthcare, science and the media and who had expertise in NCD or health reporting were identified as potential users, approached directly or recruited using the snowball principle, i.e. they were suggested by people who had already been interviewed [15]. A definition and examples of the analysed user groups can be found in the appendix (Annex Table 1). Once content was repeated by different participants across the interviews, saturation of the content was assumed and the data collection terminated [14, 17]. From the moment that no new thematic aspects for answering the research question were identified in the interview statements, no further participants were recruited. A total of 21 persons from the RKI network were contacted, 13 of whom agreed to take part in an interview. The interviews were conducted by a research associate with clinical research experience via video conference between October 2022 and January 2023 and recorded using software (Webex by Cisco [18], Audacity® [19]). A total of one hour was scheduled for each interview including information and consent. After each interview, information on the interview situation was documented. The audio files were transcribed verbatim and anonymised.

**Key statements**

► There should be a central platform which gathers, visualises and contextualises information on NCD.
► Up-to-date and reliable results on NCD are needed.
► Frequencies, determinants, care and consequences of NCD should be the content-related focus of NCD surveillance.
► Results on population groups affected by health inequalities and at a small-scale regional level are needed.
► Options for action are desired by user groups.

### 2.2 Analysis and coding process

A computer-assisted and structuring qualitative content analysis according to Kuckartz [20] was conducted. First of all, main categories were developed a priori, considering the research question and the interview guideline, that were used to code the text passages in the interviews. Subcategories were developed inductively by comparing text passages within the main categories. This procedure was followed until a category system with main and subcategories was developed and all text passages were coded. The text passages were checked several times and the category system was adapted during the course of the analysis by four research associates. For the coding, the software MAXQDA [21] was used.

**Figure 1:**
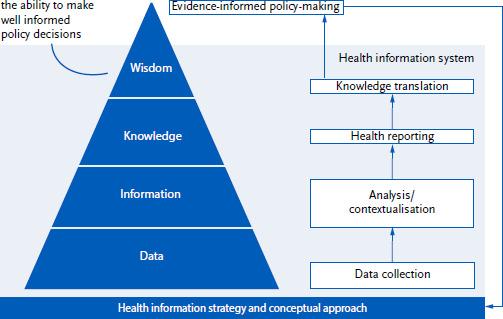
Population health monitoring model by Verschuuren and van Oers. Source: Verschuuren & van Oers [22]

To interpret the results of the content analysis, the *population health monitoring* model by Verschuuren and van Oers [22] was applied (Figure 1). This model combines the ‘information pyramid’ with the surveillance activities in order to identify the requirements of users at different levels. This pyramid is an ascending hierarchy (data-information-knowledge-wisdom), with each level representing an increasing grade of processing and contextualisation of information. The activities comprise the step-by-step process of a health information system (from data collection, analysis and contextualisation, health reporting, knowledge translation), which reaches the top of the information pyramid (with the desired goal of evidence-informed policy making). Health-related data from various sources form the basis, such as survey and examination data, register data or claims and documentation data. In order to reach the next level, data must be processed into *information*, e.g. in the form of indicators or as comparative and trend analyses. In the next stage, *knowledge* is generated by analysing information more comprehensively from a policy and practice-relevant perspective and placing it in context. In this way, explanations for observed epidemiological trends and patterns are supposed to be offered. At the highest level, the aim is to present *wisdom*. For surveillance, this concept means that evidence-based recommendations are derived from interpretations and explanatory approaches [10].

## 3. Results

### 3.1 Study participants

A total of 13 interviews were conducted with persons from the four user groups of health politics, healthcare, science and the media (Table 1).

### 3.2 Results of the qualitative content analysis

#### Category system

A category system was developed with four main categories, each with a different number of subcategories (Table 2). The main categories relate to the requirements mentioned by the respondents in terms of content, format, dissemination channel and factors influencing the use of NCD surveillance information.

#### What content is relevant?

The content requirements for the dissemination of NCD surveillance could be divided into two subcategories. First, topics relevant for reporting were mentioned and second, the type of information required on the topics.

#### Relevant topics for reporting

Mostly, users focussed on diseases and health problems. Diabetes and cardiovascular diseases were frequently mentioned, but it was emphasised that the entire spectrum of diseases is of interest. In addition to mapping new developments in population health, it is important to present the health situation of different groups of the population in order to assess the distribution of diseases. In particular, population groups that are sometimes more affected by health inequalities, such as children and adolescents or people with a history of migration, were mentioned by the interviewees. Differentiating is important for identifying approaches for preventive measures:

*‘So not only the overall figures, but basically also the explanations or the differentiation of where we should start if we want to prevent diseases or improve or do things differently in healthcare.’ (Health politics (P.) 2, position (Pos.) 9)*

Determinants of health were also emphasised as a relevant topic. Interviewees from science and healthcare emphasised that wider determinants of health situation is relevant as a supplement to behavioural risk factors to obtain a complete picture of living conditions and risk constellations:

*‘So, we also need to think about the circumstances and not just look at behaviour. […] This is essential for the NCD topic.’ (Healthcare (HC.) 2, pos. 36)*

In addition, information on healthcare and the consequences of diseases is often needed. With regard to the latter, individual (e.g. complications or functional limitations) and societal consequences (e.g. illness-related costs) were mentioned. With regard to healthcare, interest was expressed in information on treatment options. In particular, the respondents from the field of health politics emphasised the close link between the categories when it comes to assessing the impact of prevalence on the health of the population:

*‘[…] In fact, most reasons to search always deal in some way with the question of how widespread a disease is. […] And closely associated with this is information and data on the burden of disease, i.e. on the consequences of the disease, on individual health restrictions, on the need for healthcare, on the illness-related costs and so on.’ (P. 1, pos. 11)*

#### Type of content

The interviews also addressed the requirements regarding the type of content in terms of the pyramid structure. Epidemiological parameters were the most frequently mentioned type of content, followed by options for action, interpretation and the provision of raw data. The respondents also wished for a stratification according to gender, age, region and social determinants. The regional presentation, particularly at state level, was emphasised, but the need for smaller-scale analyses was also mentioned. For users from the field of politics, it is clear that the availability of regional data at federal, state, district and local authority level is an important requirement for identifying needs for action:

*‘Then you need the possibility of a general, aggregated representation, but also the possibility of going down to a smaller scale, for example for the municipalities or the federal states, to see what is happening there on the local level. Because, at the end of the day, very differentiated action will have to be taken locally.’ (P. 2, pos. 25)*

The presentation of time trends for key figures such as incidence and mortality needed to assess current developments was also discussed. The information on time trends is intended to provide an indication of how relevant a specific disease is and whether it poses a risk to the population. In addition to descriptions of key figures, the interviewees repeatedly pointed out that contextualisation and interpretation of the data is essential for a better understanding:

*‘[…] that we also add scientific evidence to explain why we see gender differences? Or why do we see an upward or downward trend or regional differences […]?’ (HC. 2, pos. 18)’.*

Finally, a need for options for action was the second most frequently mentioned requirement in terms of content. This need was mentioned several times, particularly in the science, healthcare and health politics groups. For example, the interviewees wanted potential recommendations to be provided, e.g. a list of specific measures:

*‘The question is always what actions should follow from this.’ (P. 2, pos. 50)*

#### How should the results be presented?

The interviewees’ perspectives on the presentation of content in different formats were also asked. Frequently discussed aspects were length and depth of detail, with both condensed, short formats and longer, more detailed formats being considered useful. Short formats were considered valuable as they offer a quick thematic introduction:

*‘Especially if the [information] comes from authorised sources, […] compact, with little content and a relatively small scope, it gives a good overview of the current state of knowledge with the things you should know about a particular topic. This to the format, which I also find very, very helpful.’ (Science (S.) 3, pos 13)*

Just under half of the respondents also consider longer, more detailed reports to be a relevant source of information due to the more comprehensive description of the health situation. Texts, graphics and tables are named as important elements for the presentation, although these should also be designed depending on the user group:

*‘Well, for the professional community, I think it can all be a bit tighter and denser and include classic graphics and maps, and tables too.’ (G. 2, item 28)*

For politics, the focus is more on the graphically appealing presentation, accompanied by explanatory texts. Such a format can be used without consulting other sources and offers quick and easy access:

*‘So the most valuable format is certainly a clear website or a combination of short, crisp data that can also be visualised, together with short supplementary text blocks [*…*].’ (P. 1, pos. 13)*

Graphics are regarded as an essential element for the presentation of results and should be clear and comprehensible. The labelling and design of a graph should be meaningful and functional and should not, for example, include an inappropriate scale in the axis labelling or unnecessarily different colours. Tables are mentioned by scientists as an important presentation, especially in formats such as Excel, which allow them to analyse the data themselves. Most interviewees tended to use digital formats, as these can be accessed flexibly regardless of location. Extensive reports can be quickly searched digitally by keywords. There are isolated cases where printed publications are perceived as more suitable for understanding complex contexts. The advantage of digital formats is particularly evident in the use of dashboards and interactive elements that offer users the opportunity to customise their settings:

*‘And what I just mentioned, visualisations are always very helpful, especially if you can work on them when they are flexible and dynamic.’ (P. 1, pos 17)*

Infographics, storytelling approaches and videos are also cited as other useful formats that can make it easier to absorb information:

*‘And what I also feel is essential is that we actually incorporate stories. So, even if storytelling is sometimes already such a burnt term, but still weave in a level that offers the opportunity to explain why things are the way they are and where the problems lie, because this is not always apparent from the data.’ (HC. 2, pos. 36)*

#### How does the information reach the users?

With regard to the channel through which users find information, centralised access to the results of NCD surveillance, which serves as a kind of ‘gateway’ to all important topics, was mentioned particularly frequently. The platform should also include further information to illustrate the complexity of NCD and their consequences. Less frequently, direct dialogue with scientists as a result of better networking of various stakeholders on NCD was considered important. A few interviewees perceived knowledge transfer via ‘notifications’ e.g. through social media or emails, as an incentive to delve deeper into certain topics.

#### What factors facilitate and inhibit use?

Factors that facilitate and inhibit the use of information were identified in the interviews. In all user groups, trustworthiness was important for researching and reusing content. Trustworthiness refers on the one hand to the publishing institution or the authors as well as to the standards of academic work:

*‘An independent source that is as reputable as possible is crucial for obtaining data [*…*].’ (Media (M) 1, item 13)*

Secondly, trustworthiness relates to the data basis, meaning that the origin of the data, the methodological analysis and corresponding limitations should be presented transparently. The publication of metadata and versioning of information that is frequently updated are considered helpful. When interpreting data and highlighting options for action, it is considered important to indicate the robustness of statements and their evidence base. The timeliness and relevance of information proved to be decisive factors:

*‘When I realise that information is a few years old, I often check it or discard it.’ (M. 1, pos. 25)*

Accessibility and findability of content were also mentioned as important criteria. Comprehensibility was cited less frequently as an important factor for the use of information. Standardised terminology with consistent concepts can support comprehensibility.

### 3.3 Evaluation of the diabetes surveillance formats

**Figure 2:**
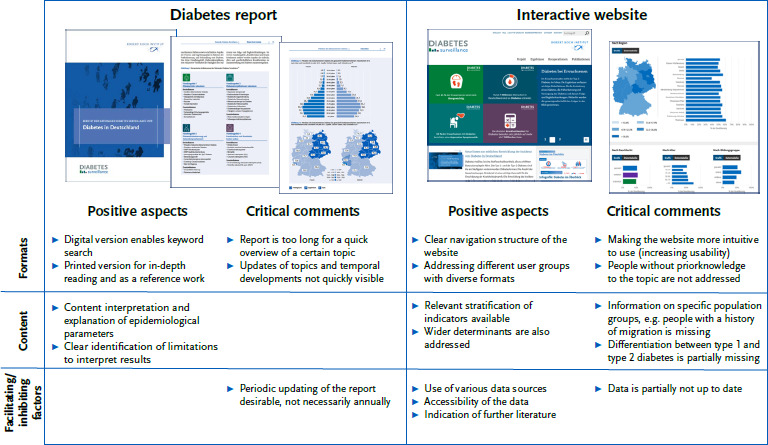
Evaluation of the existing formats of the diabetes surveillance: Positive aspects and critical comments in relation to the diabetes report and the website

A total of 11 participants who had experience with the diabetes surveillance formats were asked about their assessments of the diabetes report and the website. These were evaluated using the defined main categories and positive aspects and critical comments were identified (Figure 2). With regard to the report, for example, it was positively emphasised that results were interpreted and changes in the indicators over time were explained. For this reason, there is a desire for the report to be updated regularly (not necessarily annually). With regard to the website, for example, the range of formats used to address different user groups was mentioned positively. However, users would like to see the content expanded, e.g. on the topic of diabetes and history of migration or information on other NCD.

## 4. Discussion

This study analysed the requirements of users from science, health policy, healthcare and the media for the dissemination of NCD surveillance results. The frequency of diseases, their influencing factors, healthcare and consequences were central themes. Wider determinants of the health situation were also mentioned. While the requirements regarding the preparation of content differed according to user groups, there were similarities with regard to factors that facilitate and inhibit the use of NCD surveillance information.

### 4.1 Requirements for the dissemination of NCD surveillance

The requirements were categorised – based on the information pyramid (Figure 1) – as ‘(raw) data’, ‘information’, ‘interpretation’ (knowledge) and ‘options for action’ [22]. The lowest level contains data, particularly interesting to scientists. In addition to easy access to data, the transparent documentation and publication of metadata is important. Both aspects are also mentioned in a review article regarding factors that facilitate the use of epidemiological data [23]. Visual tools are increasingly being used to display data and during the COVID-19 pandemic, many institutions worldwide have developed dashboards to communicate case numbers [24]. However, the use of these tools requires a high level of competence in handling epidemiological data and the data must be interpreted by the user [25].

For users from politics and healthcare, the focus is more on the level of information and knowledge, in particular on the description and contextualisation of the results. In line with the present study, an interview study from the UK revealed that decision-makers in health politics often lack the capacity to analyse and evaluate data, and therefore require an interpretation of the results. Clear and unambiguous formulations are important here so that information can be quickly grasped [26]. As mentioned in the interviews, images and interactive visualisations can support the transfer of knowledge. In contrast to a dashboard, however, specifically selected illustrations to accompany the text are helpful here [27]. In an interview study from the USA, users from politics also preferred a compact one-page text summary with illustrations or infographics [28]. However, according to an Australian study, more comprehensive reports and specialised articles are also relevant, as these can present more complex issues in a nuanced way [29].

In line with the present results, both the British study and the interview studies in the USA and Australia showed the need for options for action, since potential solutions are highly relevant for people from politics and healthcare [26, 28, 29]. In this context, systematic reviews are cited as a valuable source for deriving options for action. Public health decision-makers in the USA wanted short and understandable summaries of reviews produced by trustworthy institutions [30].

In the present study, trustworthiness was one of the most important factors for the use of NCD surveillance results and was also mentioned as an important factor in a systematic review on the facilitating and inhibiting factors for the use of evidence in politics [9]. Furthermore, almost all participants stated that in addition to easy access and the comprehensibility of the content presented the timeliness and relevance of information are decisive for usage, analogous to two reviews on the topic of dissemination of scientific findings [9, 31]. Increased cooperation and regular dialogue with political stakeholders can have a supportive effect in this regard [9]. For example, knowledge brokering e.g. in interactive workshops [31] could possibly facilitate the use of surveillance results and could be included in a dissemination strategy.

### 4.2 Evaluation of diabetes surveillance formats

Overall, the interviewees rated the diabetes surveillance products as positive, and many many requirements requirements for NCD surveillance that emerged in the interviews are already being met. For example, a review on the effectiveness of measures to increase physical activity in adolescents was published presenting options for action [32].

However, additional content requirements were mentioned, e.g. information on diabetes in people with a history of migration. A study on this topic has now been published [33]. The fact that some of the indicators are not sufficiently up-to-date is emphasised as an inhibiting factor for data use. With the establishment of the population-based panel ‘Health in Germany’ at the RKI [34] and the use of secondary data, for example from the Health Data Lab [35] the latest results for important indicators of public health surveillance for NCD should be available in future. In the course of this, health monitoring is also expanded to include other NCD besides diabetes and the dialogue on the dissemination of results with important stakeholders that was started as part of diabetes surveillance is to be continued.

Some limitations must be mentioned with regard to the study conducted: The recruitment of interview participants was carried out considering various user groups that are the focus of the RKI. It should therefore be noted that most of the participants were already in contact with the RKI and use RKI results in their work. It cannot be ruled out that other people, who were not already in contact with the RKI, would have answered differently. The number of participants in the user groups also varies. For example, the media and health politics groups were smaller in the sample than the science and healthcare groups. The intercoder agreement was not checked with percentage agreements and coefficients, but the formation of categories and their assignment to text sections was evaluated and discussed in an iterative procedure within the team. The COREQ- quality criteria were considered in the conduct of the study and the documentation of the results (Annex Table 2) [36].

### 4.3 Conclusion

The requirements for the dissemination of NCD surveillance results from the perspective of users are complex, both in terms of content and format. While users from the scientific community are more interested in the data, there is a shift in the needs of persons from politics and healthcare towards the interpretation and contextualisation of results (knowledge) and the resulting options for action. Current diabetes surveillance formats already cover many user requirements. However, the requirements are not yet sufficiently met, particularly in the area of options for action. Furthermore, access to the results should be made more intuitive and more attention should be paid to updating the results and use of new data sources. The insights gained have been incorporated into the development of a new information portal for NCD (www.gbe.rki.de), which integrates contents of the diabetes surveillance and that will be evaluated and developed in terms of content and formats. Further studies will be useful to gain a more detailed insight into the specific requirements of individual user groups.

## Figures and Tables

**Table 1:** Description of the study population (n = 13)

Variables		Quantity
User groups	Health politics	2
	Media	2
	Science	5
	Healthcare	4
Years of experience in the current field of work	< 5 years	5
	≥ 5 years	8
Search reason	Internal event (e.g. publication)	11
	External event (e.g. enquiries)	9
Experience with diabetes surveillance	No	2
	Yes	11

**Table 2:** Category system with main and subcategories developed from theoretical considerations and the analysis of the interviews

**Content requirements** Topic Diseases and health problems Population groups Health determinants Healthcare and consequences of diseases Type of content Raw data Epidemiological parameters/information Interpretation/explanations/contextualisation of content Options for action
**Requirements for the format** Target group specificity (type and manner of presentation) Length and depth of detail Elements for presenting content Digital vs. print Interactivity Storytelling/video reports
**Channel (knowledge transfer)** Direct exchange Notification Central infrastructure
**Facilitating and inhibiting factors of use** Trustworthiness Timeliness/Relevance Findability/Accessibility Comprehensibility Consistency/comparability

**Annex Table 1:** Definition and examples of the analysed user groups

User group	Definition of	Examples
Politics	Stakeholders, bodies with political responsibility that require expert advice on prevention and health promotion, health protection and disease control for the population.	Employees of the ministries of health at federal and state level Political office holders at federal, state and municipal level
Healthcare and public health practice	Individuals, groups and institutions whose professional focus is on preventing, recognising and coping with diseases	Employees of the public health service, professional associations, scientific societies from the healthcare sector
Science	Individuals and institutions that systematically search for and share scientific knowledge with the aim of making decisions that protect and improve health.	Employees at universities, colleges and scientific institutes such as the Helmholtz Association, the Leibniz Association or the Max Planck Society
Press	Editors and freelancers of (daily) newspapers, magazines, radio/TV and online services.	Journalists from (daily) newspapers (e.g. Bild, FAZ, TAZ), magazines (e.g. Spiegel, Apothekenumschau) or radio and television (e.g. ARD, ZDF).

**Annex Table 2:** COREQ (COnsolidated criteria for REporting Qualitative research) Checklist. Quelle: Tong et al. 2007 [36]

Topic	Item No.	Guide Questions/Description	Reported on Page No.
**Domain 1: Research team and reflexivity**			
*Personal characteristics*			
Interviewer/facilitator	1	Which author/s conducted the interview or focus group?	Page 3
Credentials	2	What were the researcher’s credentials? E.g. PhD, MD	Page 3
Occupation	3	What was their occupation at the time of the study?	Page 3
Gender	4	Was the researcher male or female?	-
Experience and training	5	What experience or training did the researcher have?	Page 3
*Relationship with participants*			
Relationship established	6	Was a relationship established prior to study commencement?	Page 8
Participant knowledge of the interviewer	7	What did the participants know about the researcher? e.g. personal goals, reasons for doing the research	Page 3
Interviewer characteristics	8	What characteristics were reported about the interviewer/facilitator? e.g. Bias, assumptions, reasons and interests in the research topic	Page 3/8
**Domain 2: Study design**			
*Theoretical framework*			
Methodological orientation and Theory	9	What methodological orientation was stated to underpin the study? e.g. grounded theory, discourse analysis, ethnography, phenomenology, content analysis	Page 3
*Participant selection*			
Sampling	10	How were participants selected? e.g. purposive, convenience, consecutive, snowball	Page 2
Method of approach	11	How were participants approached? e.g. face-to-face, telephone, mail, email	Page 3
Sample size	12	How many participants were in the study?	Page 3
Non-participation	13	How many people refused to participate or dropped out? Reasons?	Page 3
*Setting*			
Setting of data collection	14	Where was the data collected? e.g. home, clinic, workplace	Page 3
Presence of non-participants	15	Was anyone else present besides the participants and researchers?	Page 3
Description of sample	16	What are the important characteristics of the sample? e.g. demographic data, date	Page 4
*Data collection*			
Interview guide	17	Were questions, prompts, guides provided by the authors? Was it pilot tested?	Page 2
Repeat interviews	18	Were repeat interviews carried out? If yes, how many?	-
Audio/visual recording	19	Did the research use audio or visual recording to collect the data?	Page 2
Field notes	20	Were field notes made during and/or after the interview or focus group?	Page 2
Duration	21	What was the duration of the interviews or focus group?	Page 2
Data saturation	22	Was data saturation discussed?	Page 2
Transcripts returned	23	Were transcripts returned to participants for comment and/or correction?	-
**Domain 3: analysis and findings**			
*Data analysis*			
Number of data coders	24	How many data coders coded the data?	Page 3
Description of the coding tree	25	Did authors provide a description of the coding tree?	Page 4
Derivation of themes	26	Were themes identified in advance or derived from the data?	Page 3
Software	27	What software, if applicable, was used to manage the data?	Page 3
Participant checking	28	Did participants provide feedback on the findings?	-
*Reporting*			
Quotations presented	29	Were participant quotations presented to illustrate the themes/findings? Was each quotation identified? e.g. participant number	Page 4–6
Data and findings consistent	30	Was there consistency between the data presented and the findings?	Page 4–7
Clarity of major themes	31	Were major themes clearly presented in the findings?	Page 4–7
Clarity of minor themes	32	Is there a description of diverse cases or discussion of minor themes?	Page 4–7
